# Is Behçet’s syndrome associated with an increased risk of ischemic heart disease? A real-world evidence in Taiwan

**DOI:** 10.1186/s13075-021-02543-6

**Published:** 2021-06-04

**Authors:** Chun-Yu Lin, Hung-An Chen, Chun-Hsin Wu, Yu-Jih Su, Tsai-Ching Hsu, Chung-Yuan Hsu

**Affiliations:** 1 Division of Rheumatology, Department of Internal Medicine, National Cheng Kung University Hospital, College of Medicine, National Cheng Kung University, No.138, Sheng Li Road, Tainan, 704 Taiwan; 2 Division of Allergy-Immunology-Rheumatology, Department of Internal Medicine, Chi Mei Medical Center, Tainan, Taiwan; 3 Chia Nan University of Pharmacy and Science, Tainan, Taiwan; 4 Department of Internal Medicine, National Cheng Kung University Hospital, College of Medicine, National Cheng Kung University, Tainan, Taiwan; 5 Division of Rheumatology, Allergy, and Immunology, Department of Internal Medicine, Chang Gung Memorial Hospital, Kaohsiung, Taiwan; 6 College of Medicine, Chang Gung University, Taoyuan, Taiwan; 7 Institute of Biochemistry, Microbiology and Immunology, Chung Shan Medical University, Taichung, Taiwan; 8 Division of Rheumatology, Allergy and Immunology, Department of Internal Medicine, Kaohsiung Chang Gung Memorial Hospital, Chang Gung University College of Medicine, No. 123, Ta Pei Road, Niao Sung District, Kaohsiung, 83301 Taiwan

**Keywords:** Behçet’s syndrome, Ischemic heart disease, Long-term mortality

## Abstract

**Background:**

A variety of chronic inflammatory diseases are linked to ischemic heart disease (IHD); however, this association is less well studied in patients with Behçet’s syndrome (BS). The primary objective of this study was to examine the impact of BS on the risk of IHD. The secondary objective was to estimate the long-term mortality risk in patients with BS.

**Methods:**

Using a retrospective cohort design based on the Taiwan National Health Insurance Database, patients diagnosed with BS between 2000 and 2013, without prior history of IHD, were compared to non-BS individuals. The BS and non-BS cohorts were matched with a 1:2 ratio by propensity score, accounting for the following confounders: age, sex, year of index date, comorbidities, and drug exposure. Cox proportional hazard regression was used to derive the hazard ratio (HR) for IHD and mortality. The long-term survival rate was estimated using the Kaplan-Meier method.

**Results:**

After propensity score matching, a total of 1554 patients newly diagnosed with BS and 3108 control subjects were identified. The incidence rate of IHD in the BS and control groups was 2.7 and 2.9 per 1000 person-years, respectively. The risk of IHD was comparable between BS and control cohorts [adjusted HR, 1.03; 95% confidence interval (CI), 0.66 to 1.62]. The 5- and 10-year survival rate of BS patients was 96.8% and 95.0%, respectively. Patients with BS exhibited a significantly higher risk of mortality than the sex- and age-matched general population (adjusted HR, 1.73; 95% CI, 1.30 to 2.32).

**Conclusion:**

Unlike other chronic systemic autoimmune disorders, BS does not appear to be associated with an excess risk of IHD.

## Introduction

Behçet’s syndrome (BS) is a multi-systemic auto-inflammatory disorder characterized by recurrent oral and/or genital ulcerations, uvea inflammation, and vasculitis [1–3]. Epidemiological studies have shown that the incidence and prevalence of BS varies across countries and geographic areas, being highest in the Mediterranean countries and the Far East area [2–4]. The clinical features and expressions of BS also exhibit geographic variation. For example, the involvement of the gastrointestinal tract is more frequent among patients with BS in Asia than in Turkey [5, 6]. Regarding the survival of patients with BS, some studies have been performed in the last century, mostly in Turkey [7]. Recently, the mortality risk for patients with BS was reported by a study in the United Kingdom (UK) [8]. Despite the geographical variation in the clinical presentation of BS, it is uncertain whether the long-term prognosis of BS also differs across different ethnic populations. Moreover, few studies have addressed the long-term mortality risk of BS in the Chinese population at a general population level.

A number of chronic inflammatory disorders, such as rheumatoid arthritis (RA), systemic lupus erythematosus (SLE), and idiopathic inflammatory myopathy, have been strongly linked with atherosclerosis and ischemic heart disease (IHD) in recent decades [9–11]. Systemic inflammation has been recognized as a key player in the initiation and progression of atherosclerotic heart disease in patients with chronic autoimmune diseases [10]. Although major vascular involvement is far more common in the disease course of BS, arterial lesions usually manifest as pulmonary arterial aneurysms, whereas coronary artery disease has not been recognized as a prominent feature of BS in earlier clinical studies [12, 13]. Moreover, a study assessing subclinical atherosclerosis in the carotid and femoral arteries has shown that the frequency of atherosclerotic plaques is comparable between patients with BS and healthy control subjects [14]. Another study using computed tomography to investigate the condition of the coronary artery found a relatively low frequency of coronary artery calcification in male patients with BS, even in those with diffuse large vessel disease [15]. Therefore, whether the risk of IHD is as high in BS as in other chronic inflammatory diseases remains unclear.

We conducted this large-scale cohort study to determine whether or not the risk of clinically significant IHD was elevated in patients with BS compared to that in the non-BS population. We also sought to determine the long-term survival rate of BS patients and compare their mortality risk with that of matched individuals without BS.

## Methods

### Study design and data source

**Fig. 1:**
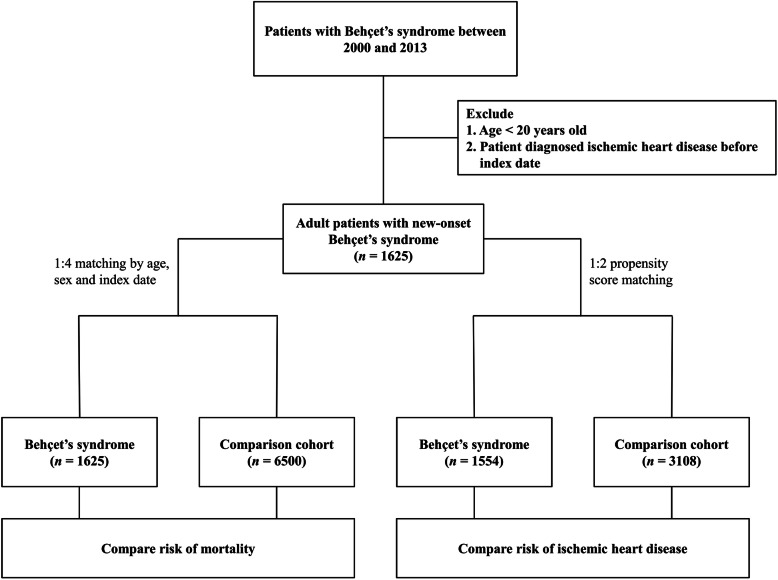
Flow chart of the study procedure

This retrospective follow-up study was conducted using the National Health Insurance Research Database (NHIRD) in Taiwan. A mandatory National Health Insurance (NHI) program was launched in Taiwan in 1995, and over 99% of the population had been enrolled in this program until 2013. The NHI program provides broad health care coverage, including ambulatory care, inpatient care, dental care, prescription drugs, and surgical procedures, for Taiwan’s residents. Thus, NHIRD, which was constructed based on the NHI program and contains detailed health care data, is one of the largest databases worldwide and is used extensively for epidemiological and long-term follow-up studies [16, 17]. The identification number of patients in NHIRD is encrypted to ensure privacy protection. Therefore, informed consent in our study was waived, and the National Cheng Kung University Hospital Institutional Review Board approved the study protocol (A-EX-109-017). The study flow chart of the selection process of patients and comparison cohort is summarized in Fig. 1.

### Study cohort

Adult patients (aged > 20 years) with BS were identified during the period from January 1, 2000, to December 31, 2013, using the International Classification of Diseases, Ninth Revision, Clinical Modification (ICD-9-CM) code 136.1, from outpatient or inpatient records in the NHIRD. To ensure the accuracy of the diagnosis, patients who were considered to have BS were also required to hold a catastrophic illness certificate for BS, in addition to having corresponding diagnostic codes. Patients with severe disorders, such as cancer, autoimmune diseases, end-stage kidney disease requiring dialysis, or type 1 diabetes, can apply for a catastrophic illness certificate under the Taiwan NHI system. Patients who were issued such a certificate may be exempted from copayment when seeking medical care related to such illness. To obtain this certificate for BS, medical records, laboratory results, and image findings are reviewed by two board-certified rheumatologists, and the International Study Group (ISG) criteria for BS should be fulfilled [18]. Patients without a certificate were excluded from our study, ensuring proper identification of BS.

The index date was defined as the date of first diagnosis of BS. We also screened the 2-year period before the index date to ensure that no diagnosis of BS had been made during that period, and we only included incident cases of BS in our study. Patients who developed IHD before the index date were also excluded from the study.

### Comparison (control) group

Patients without BS were selected from the subset of NHIRD identified between 2000 and 2013. The index dates for the non-BS cohort were randomly assigned, corresponding to the distribution of those in the BS cohort. The exclusion criteria for the BS cohort were also applied to the non-BS cohort; i.e., patients aged < 20 years or experiencing IHD before the index date were not included in the control group.

### Assessment of covariates

Demographic information, including age and sex, was retrieved from the database. Comorbid diseases were searched in the 2-year period before the index date. Patients were designated as having a specific comorbidity if they had received corresponding diagnostic codes at least one time in the inpatient claims or at least three times in the outpatient claims. Comorbidities analyzed in the present study were as follows: diabetes (ICD-9-CM code 250), hypertension (ICD-9-CM codes 401-405), dyslipidemia (ICD-9-CM code 272), chronic obstructive lung disease (COPD; ICD-9-CM codes 491, 492, 496), and chronic kidney disease (ICD-9-CM codes 580-588). Exposure to various medications, including non-steroidal anti-inflammatory drugs (NSAIDs), systemic corticosteroids, statins, and aspirin, was also recorded within the 90-day period of the index date.

### Study outcomes

The main outcome of this study was the occurrence of IHD. Development of IHD was ascertained by ICD-9-CM codes 410 (acute myocardial infarction) and 411 (acute and subacute forms of ischemic heart disease) in the principal diagnosis of inpatient claims. The accuracy of the diagnosis of acute myocardial infarction was validated in Taiwan’s NHIRD with a positive predictive value of 0.93 when using only the principal diagnosis [19]. When analyzing the main outcome, patients were followed up until the development of IHD, death, or the end of the study (December 31, 2013), whichever occurred first. The secondary outcome was mortality. When analyzing the secondary outcome, patients were followed up until death or the end of the study (December 31, 2013), whichever occurred first.

### Process of propensity score matching in the assessment of IHD

We carried out propensity score matching for analyzing the risk of IHD in the BS versus non-BS group, in order to reduce the confounding effects of age, sex, comorbidities, and medication use on the outcome. We constructed a logistic regression model with patients having BS or not as the dependent variable and age, sex, year of index date, diabetes, hypertension, COPD, hyperlipidemia, use of stains, aspirin, corticosteroids, and NSAIDs as independent variables, in order to estimate the propensity score. Then, each patient with BS was matched to two subjects without BS using a caliper width of 0.2 of the pooled standard deviation of the logit of the propensity score. The quality of balance of covariates between BS and non-BS groups was checked by the mean standardized difference. If this difference was less than 0.1, the balance of confounding variables was considered to be acceptable [20].

### Matching for age, sex, and year of index date in the assessment of mortality

For estimating the mortality risk in patients with BS compared with that in the general population, we constructed another comparison control cohort, which was matched with the BS cohort with a 1:4 ratio (Fig. 1). The matching variables in this analysis included age, sex, and year of index date.

### Power and statistical analysis

The study design assumed an IHD event rate of 3% in the non-BS group at 10 years’ follow-up, and a hazard ratio (HR) of 2.0 in the BS group versus non-BS group. Then, the sample size was calculated with the use of a two-sided test at the 0.05 significance level and the 90% power level. It was determined that 827 patients with BS and 1652 controls should be included in the current study.

Continuous and categorical variables were presented as mean ± standard deviation and percentage, respectively. The Kaplan-Meier method was used to calculate the cumulative incidence of IHD and the survival rate in the BS and matched control cohorts. A log-rank test was used to determine whether a significant difference existed with respect to the incidence of IHD and survival between BS and non-BS groups. The doubly robust method, combining the regression model for outcomes and propensity score models, was used to derive the final effect estimates of BS on the IHD incidence [21, 22]. The advantage of this method was that the estimates were unbiased as long as either model (propensity score or outcome regression) was correctly specified. Specifically, we used a Cox proportional hazard regression model adjusting for sex, age, comorbidities, and medications to derive the impact of BS on IHD occurrence after propensity score matching of the BS and control groups. We further performed a sensitivity analysis using a Fine and Gray competing risk regression model [23], considering the event of death as a competing event when analyzing the primary outcome of IHD occurrence. The threshold of the significance level was set at a two-sided *P* value < 0.05. All data management, graph preparation, and statistical analyses were performed using Stata version 13 software (StataCorp, College Station, TX, USA).

## Results

A total of 1625 patients with BS were identified in the period from 2000 to 2013. Table 1 presents the demographic information, baseline characteristics, and medication exposure in the BS and control cohorts. After propensity score matching, 1554 patients with BS and 3108 matched control subjects remained for further analysis in our study. The mean standardized difference of all variables between the BS and control groups after matching was less than 0.1. Female sex accounted for 58% of the BS cohort. The mean age of patients with BS was 39.2 ± 12.0 years. The most prevalent comorbid disease in patients was hypertension, followed by hyperlipidemia, diabetes, chronic kidney disease, and COPD. However, the percentage of these comorbidities was less than 10% in all cases. One third of patients with BS had received corticosteroid treatment, while statin and aspirin were less prescribed.

**Table 1:** Summary of demographics and characteristics of patients with Behçet’s syndrome and controls without Behçet’s syndrome

Characteristics	Before propensity score matching	After propensity score matching		
	Behçet’s syndrome (n = 1625)	Behçet’s syndrome (n = 1554)	Control (n = 3108)	Standardized mean difference
Sex, n (%)				0.04
Female	942 (58.0)	901 (58.0)	1735 (56.0)	
Male	683 (42.0)	653 (42.0)	1373 (44.0)	
Age group, n (%), years				0.001
18–44	1131 (69.6)	1064 (68.5)	2130 (68.5)	
≧45	494 (30.4)	490 (31.5)	978 (31.5)	
Age, mean ± SD, years	38.8 ± 11.9	39.2 ± 12.0	39.1 ± 12.2	0.008
Comorbidities, n (%)				
Diabetes	39 (2.4)	39 (2.5)	88 (2.8)	-0.020
Hypertension	104 (6.4)	103 (6.6)	211 (6.7)	-0.006
Hyperlipidemia	40 (2.5)	40 (2.6)	98 (3.1)	-0.035
Chronic kidney disease	21 (1.3)	21 (1.4)	41 (1.3)	0.003
COPD	21 (1.3)	21 (1.4)	40 (1.3)	0.006
Medications, n (%)				
NSAID	465 (28.6)	405 (26.1)	802 (25.8)	0.006
Corticosteroid	589 (36.3)	520 (33.5)	1048 (33.7)	-0.005
Statin	16 (1.0)	16 (1.0)	46 (1.4)	-0.04
Aspirin	37 (2.3)	33 (2.1)	67 (2.2)	-0.002

*COPD*, chronic obstructive pulmonary disease; *NSAID*, nonsteroidal anti-inflammatory drug; *SD*, standard deviation

### Incidence and risk of IHD

**Fig. 2:**
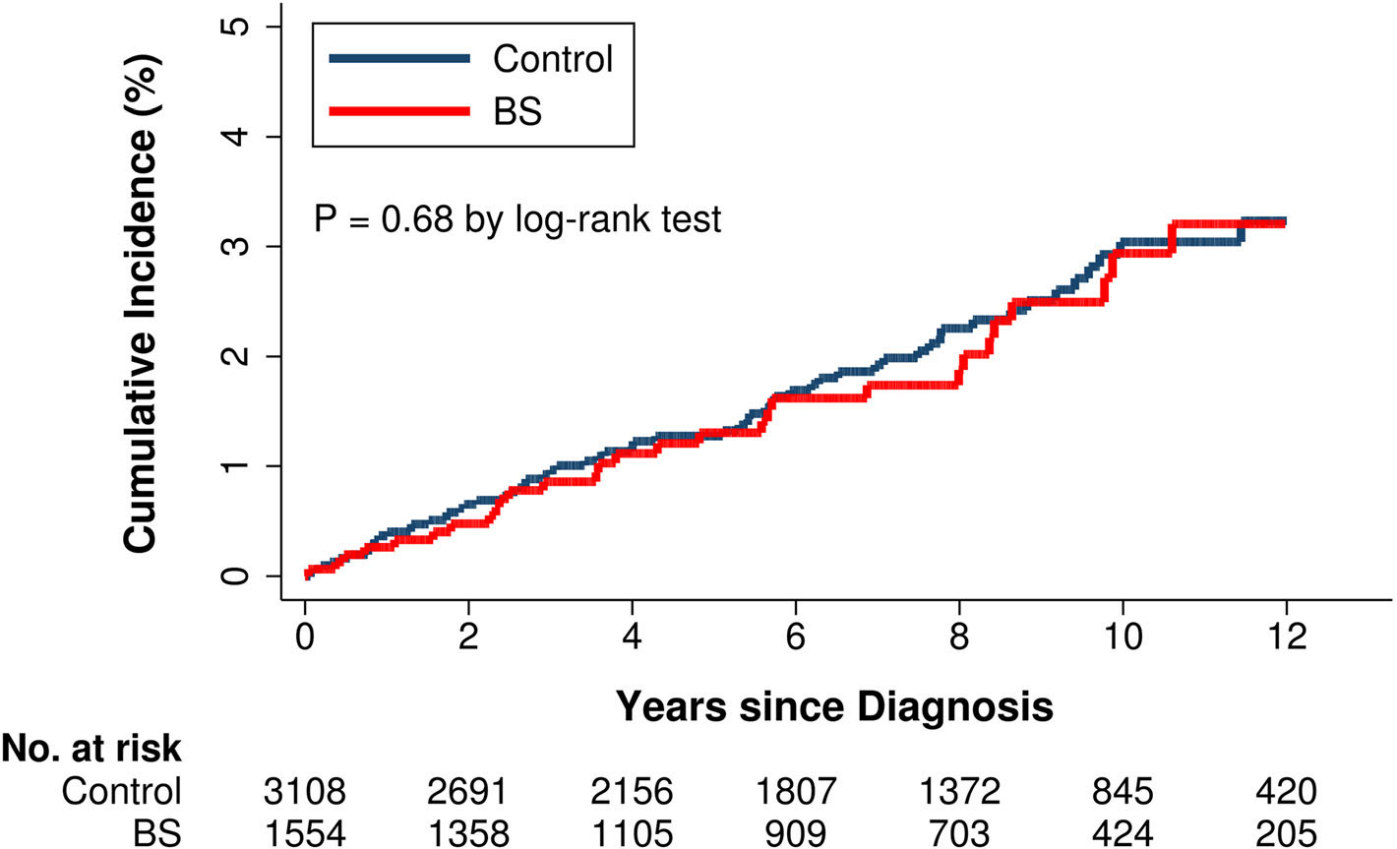
Cumulative incidence of ischemic heart disease among patients with Behçet’s syndrome (BS) and control subjects without BS after propensity score matching

Among the 1554 patients with BS and 3108 matched control subjects, 29 (1.9%) and 63 (2.0%), respectively, developed IHD during follow-up (Table 2). The incidence rate of IHD in BS and control cohorts was 2.7 and 2.9 per 1000 person-years, respectively. Figure 2 presents the cumulative incidence of IHD; no significant difference was detected between groups (log-rank test, P value = 0.68). Cox regression analysis, adjusting for age, sex, comorbidities, and drug use, revealed that the risk of IHD was similar between patients and control subjects (HR, 1.03; 95% confidence interval [CI], 0.66 to 1.62). Sensitivity analysis using a competing risk regression model also showed similar results (sub-distribution HR, 1.00; 95% CI, 0.64 to 1.57).

**Table 2:** Incidence rate and relative risk for ischemic heart disease in patients with Behçet’s syndrome and in propensity score-matched control subjects

	Behçet’s syndrome (n = 1554)	Control (n = 3108)
**Ischemic heart disease**		
Cases, n (%)	29 (1.9)	63 (2.0)
Person-years	10,909	21,554
IR (95% CI)*	2.7 (1.9–3.8)	2.9 (2.2–3.7)
aHR (95% CI)**	1.03 (0.66–1.62)	reference
sHR	1.00 (0.64–1.57)	reference

^*^Expressed per 1000 person-years

^**^Adjusted for age, sex, diabetes, hypertension, chronic kidney disease, COPD, and use of NSAIDs, corticosteroids, statins, and aspirin

*COPD*, chronic obstructive pulmonary disease; *NSAID*, nonsteroidal anti-inflammatory drug; *IR*, incidence rate; *aHR*, adjusted hazard ratio; *sHR*, sub-distribution hazard ratio; *CI*, confidence interval

### Mortality rate

**Fig. 3:**
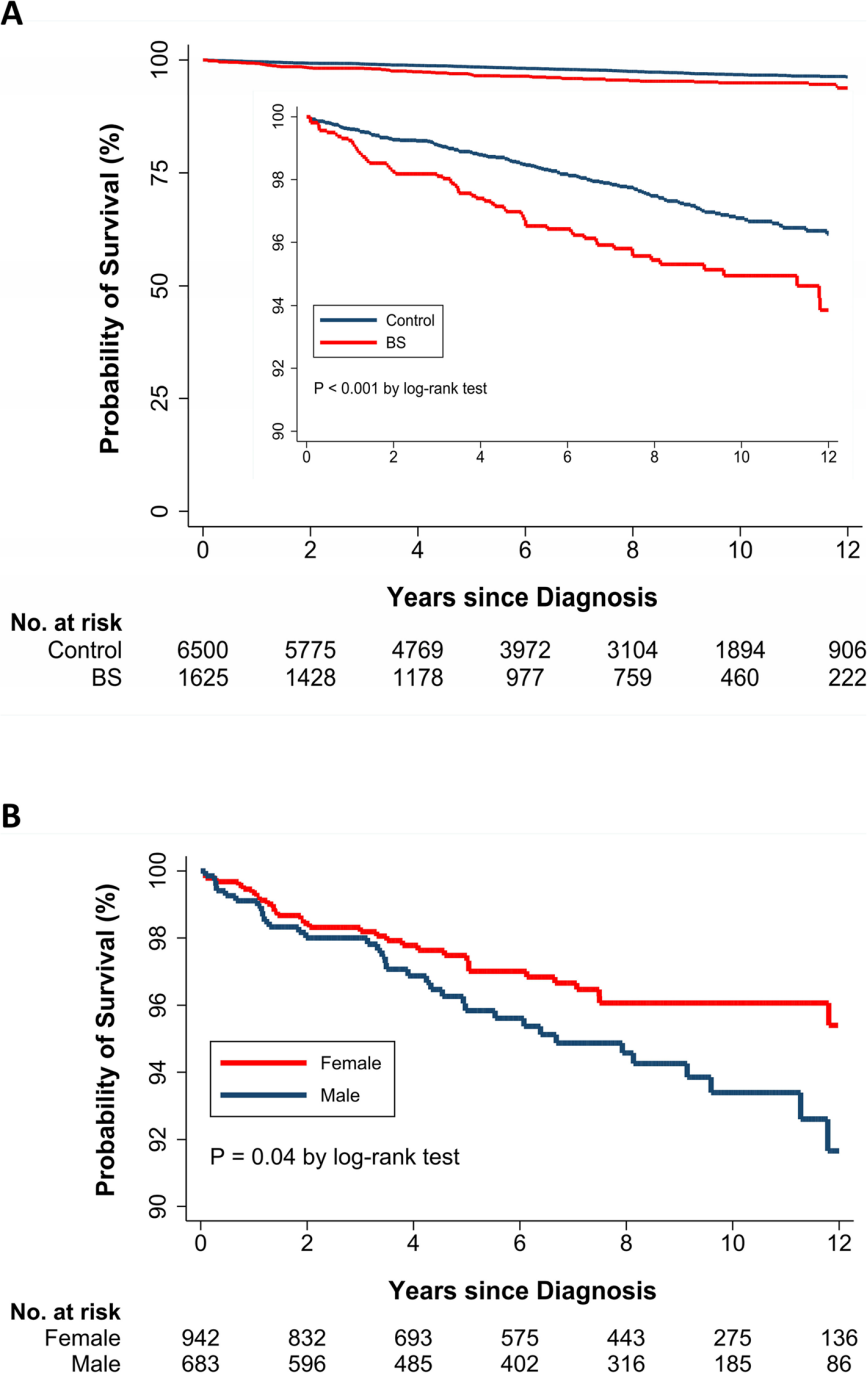
**A** Comparison of the survival rate in patients with Behçet’s syndrome (BS) and that in age- and sex-matched control subjects. **B** Survival rate in patients with BS stratified by sex

A total of 1625 patients with BS and 6500 control subjects were included in the analysis of mortality risk during the study period (Table 3). Among them, 65 (4.0%) and 157 (2.4%) subjects in the BS and control cohort, respectively, died. The rate of mortality in patients and controls was 5.60 and 3.33 per 1000 person-years, respectively. The 3-, 5-, and 10-year survival rates of patients were 98.2%, 96.8%, and 95.0%, respectively, while the respective rates in control subjects were 99.1%, 98.5%, and 96.8% (Fig. 3A). The log-rank test indicated that patients exhibited a higher mortality risk compared with control patients (P value < 0.001). Cox regression model analysis showed that the risk of death in the BS group was 73% higher than that in the sex- and age-matched control group (HR, 1.73; 95% CI, 1.30 to 2.32). In addition, the risk of death was higher for male than for female patients with BS (P value = 0.04) (Fig. 3B).

**Table 3:** Incidence rate and risk for death in patients with Behçet’s syndrome and age-, sex- and index date-matched control subjects

	Behçet’s syndrome (n = 1625)	Control (n = 6500)
**Death**		
Cases, n (%)	65 (4.0)	157 (2.4)
Person-years	11,615	47,130
IR (95% CI)*	5.60 (4.32–7.13)	3.33 (2.83–3.90)
HR (95% CI)	1.73 (1.30–2.32)	Reference

^*^Expressed per 1000 person-years

*HR*, hazard ratio; *IR*, incidence rate; *CI*, confidence interval

## Discussion

We conducted a retrospective study to assess the risk of IHD and mortality in a representative sample of patients with BS in Taiwan. To the best of our knowledge, no previous large-scale epidemiological investigation has been conducted in the Han Chinese population. The principal findings of our study were (1) that patients with BS did not exhibit a higher risk of IHD compared with the respective risk of the non-BS population; (2) that the 5- and 10-year survival rates of patients with BS were 96.8% and 95.0%, respectively, which were significantly lower than the rates of sex- and age-matched control subjects; and (3) that male sex was a risk factor of mortality in these patients.

It has been well established that a number of chronic rheumatic disorders, particularly RA and SLE, are associated with substantially increased risk of atherosclerosis and IHD, independent of classical risk factors such as diabetes, hypertension, and dyslipidemia [24, 25]. Accumulating evidence has shown that all stages of atherosclerosis and plaque formation are mainly driven by inflammatory processes [26]. Elevated levels of acute phase reactants, pro-inflammatory cytokines, and the presence of circulating autoantibodies and specific T cell subsets observed in RA and SLE lead to a higher atherosclerotic burden [25]. Moreover, a recent randomized double-blind trial revealed that colchicine, an anti-inflammatory medication, reduces the risk of ischemic cardiovascular events among patients who have experienced a recent myocardial infarction, supporting the role of inflammation in the pathogenesis of IHD [27]. Other factors contributing to IHD in patients with chronic rheumatic diseases include higher prevalence of comorbidities and higher exposure to corticosteroids and NSAIDs. However, not all chronic systemic inflammatory diseases predispose patients to acute coronary events and accelerated atherosclerosis. Some previous studies have demonstrated that the incidence of acute myocardial infarction is not elevated for patients with Crohn’s disease or ulcerative colitis [28, 29]. Moreover, several previous studies have also demonstrated that the mean intima media thickness of carotid arteries is not significantly higher in patients with BS than in healthy controls [14, 30, 31]. Our present results indicate that BS does not increase the risk of IHD, as we found a similar risk in the general population. This may be ascribed to the episodic nature of inflammation in BS [1, 3], contrary to its relatively persistent and prolonged nature in RA. In addition, the disease activity of BS tends to be attenuated with the passage of time [1, 3]. The above may contribute to the less pronounced cumulative inflammatory vascular damage in BS, as compared to that in RA or SLE. Another explanation for this differential risk of IHD is that the underlying pathophysiological mechanisms of BS are distinct from those of RA/SLE [32]. Thus, it remains to be elucidated whether chronic inflammation is a common critical determinant in the process of accelerated atherosclerosis or disease-specific inflammatory profiles that are more crucial exist.

Recently, a database-driven study in the UK also demonstrated a similar risk of IHD between patients with BS and age- and sex-matched control subjects after adjusting confounding factors in the primary analysis [8]. However, in their sensitivity analysis, which only included incident cases of BS for assessing the primary outcome, the authors reported a significantly higher risk of IHD among patients with BS. The results of this additional analysis regarding the risk of IHD were not consistent with our findings. Nevertheless, the number of incident patients with BS in the UK study was only about 400, which could influence the precision of the estimates, as evidenced by the relatively wider CI of the HR for the risk of IHD, shown in the sensitivity analysis. Furthermore, lipid-lowering agents were the only medications that were included in their regression model for estimating the risk of IHD. In contrast, in our study, we considered and adjusted the effects of other drugs that may affect the occurrence of IHD, such as corticosteroids, aspirin, and NSAIDs [33]. Additionally, in the UK study, data were extracted from an anonymized primary care database, and authors could not clinically validate the diagnosis of BS, whereas our study was based on a nationwide database including patients from general practice, tertiary care, and hospitalized patients. Another explanation for the discrepant results may be that the rate of IHD is higher in Western than Eastern counties [34].

Another study conducted in Korea using a national insurance database and enrolling a large number of patients with BS revealed that the risk of myocardial infarction was 60% higher than that in the general population [35]. However, similar to the UK study, this Korean study also did not address the impact of various medications, which may have detrimental effects on coronary vasculature. The average age of diagnosis of BS was also higher than that in our BS population. Thus, it is possible that the estimated influence of BS on IHD in the Korean study may have been exaggerated, as some confounders were not accounted for.

With respect to long-term prognosis of BS, a recent epidemiological study in the UK showed that the 10-year survival rate is approximately 92%, and male sex carries a higher risk of mortality [8]. A French study enrolling 817 patients with BS revealed that the 3-, 5-, and 10-year survival rates were 97.9%, 96.7%, and 95.7%, respectively [36]. Another study conducted in Korea using an insurance database estimated that the 5-year survival rate of patients with BS was around 98% [35]. These findings are comparable to our results.

The strength of our study lies in the use of a nation-scale database, enrolling nearly all people living in Taiwan, as well as patients from all levels of health care, to estimate the risk of IHD and mortality. The advantage of this database ensures that selection and referral bias are minimized. In addition, we used propensity score matching and a doubly robust method to account for the confounding effect of age, sex, comorbidities, and various drug usage on the outcome of interest. The diagnostic accuracy of BS in our study was high, as we only included patients with a catastrophic illness certificate. However, there were still some limitations that should be mentioned. First, some important variables regarding lifestyle, such as smoking, were not available in our analysis. However, we extracted information on the percentage of individuals with COPD, both among patients with BS and control subjects, which could be considered as a surrogate marker of smoking, at least to some extent. Second, there was no detailed information on symptoms/signs and laboratory or radiographic results in this claims-database. Thus, we could not evaluate the disease activity status for each patient with BS and analyze its influence on the outcome. Third, we did not have access to the cause of death. Therefore, we could not analyze whether the higher risk of mortality in patients was related to the major vascular manifestations of BS or due to infectious complications. Fourth, although we used validated codes to capture the primary outcome, there could be residual misclassification bias, which was an inherent limitation of the administrative claims database.

## Conclusion

Patients with BS do not exhibit excessive risk of IHD when compared to the respective risk of the non-BS population. It appears that BS cannot be viewed as an independent risk factor of IHD, which is contrary to what is observed for other chronic autoimmune diseases, such as RA or SLE. The 5- and 10-year survival rates among patients with BS were 96.8% and 95.0%, respectively, which is generally better than the rates of other systemic rheumatic diseases. However, these patients had a 1.7 times higher risk of mortality compared with the general population. Regular and cautious monitoring is still crucial in the management of patients with BS.

## Data Availability

The datasets used and/or analyzed during the current study are available from the corresponding author on reasonable request.

## Abbreviations

BS: Behçet’s syndrome
CI: Confidence interval
COPD: Chronic obstructive lung disease
HR: Hazard ratio
ICD-9-CM: International Classification of Diseases, Ninth Revision, Clinical Modification
IHD: Ischemic heart disease
ISG: International Study Group
NHI: National Health Insurance
NHIRD: National Health Insurance Research Database
NSAIDs: Non-steroidal anti-inflammatory drugs
RA: Rheumatoid arthritis
SLE: Systemic lupus erythematosus
